# Experimental Study of the Irrational Phase Synchronization of Coupled Nonidentical Mechanical Metronomes

**DOI:** 10.1371/journal.pone.0118986

**Published:** 2015-03-18

**Authors:** Zhiwen Song, Ye Wu, Weiqing Liu, Jinghua Xiao

**Affiliations:** 1 School of Science, Beijing University of Posts and Telecommunications, Beijing 100876, China; 2 School of Science, Jiangxi University of Science and Technology, Ganzhou 341000, China; Universiteit Gent, BELGIUM

## Abstract

It has recently been observed in numerical simulations that the phases of two coupled nonlinear oscillators can become locked into an irrational ratio, exhibiting the phenomenon of irrational phase synchronization (IPS) [Phys. Rev. E 69, 056228 (2004)]. Here, using two coupled nonidentical periodic mechanical metronomes, we revisit this interesting phenomenon through experimental studies. It is demonstrated that under suitable couplings, the phases of the metronomes indeed can become locked into irrational ratios. Numerical simulations confirm the experimental observations and also reveal that in the IPS state, the system dynamics are chaotic. Our studies provide a solid step toward further studies of IPS.

## Introduction

As a universal concept in nonlinear science, synchronization has been widely observed in both natural and manmade systems [1]. One well-known example is the synchronization of hand clapping after a show performance, in which audiences are found to begin clapping their hands with the same rhythm after a short transient period [2]. Additional examples of synchronization are observed in biological systems [3,4], psychiatric diseases [5,6], and many other contexts [7,8,9]. In general, synchronization refers to the coherent motion of coupled oscillators, which typically occurs when the coupling strength exceeds some critical value. Depending on the specific form of the coherent motion, synchronization can be classified into various types, including complete synchronization, phase synchronization [10,11,12], generalized synchronization, and lag synchronization [1].

Phase synchronization is defined as the locking of the phases of coupled oscillators [13,14]. Specifically, let *ϕ*_1,2_(*t*) be the instantaneous phases of the coupled oscillators; phase synchronization is said to be achieved if |*ϕ*_1_ (*t*) – (*m / n*) *ϕ*_2_(*t*)| < *c*, where *c* ∊ [0,*π*] is a constant and *m* and *n* are two integers. Because any 2*π* increment in *ϕ*_1_ is accompanied by an (*m* / *n*)2*π* increment in *ϕ*_2_, this type of synchronization is also called *m*:*n* phase synchronization. Phase synchronization is trivial when the oscillators are periodic because | *ϕ*_1_(*t*) – (*m* / *n*)| *ϕ*_2_(*t*) < *c* even in the absence of coupling, given that their natural frequencies (the frequencies of the isolated oscillators) *f*_10,20_ satisfy *f*_10_ / *f*_20_ = *m* / *n*. As such, phase synchronization is primarily of interest for chaotic systems [15,16]. Because only the phases of the oscillators are correlated, phase synchronization represents a weaker type of coherent motion than complete synchronization and is typically observed at a lower coupling strength than the latter. However, unlike complete synchronization, which requires the coupled oscillators to exhibit identical dynamics, phase synchronization is observable for nonidentical oscillators, making it more relevant to practical situations.

Previous studies of phase synchronization have been primarily focused on rational phase locking, i.e., cases in which the ratio *r* = *m*:*n* is a rational number. Recently, it has been shown numerically that under certain circumstances, the phases of two coupled chaotic oscillators can also become locked into an irrational ratio, i.e., |Δ_*r*_ *ϕ* − *c*| < π, where Δ_*r*_ *ϕ* (t) = | *ϕ*_1_ − *r ϕ*_2_| is the phase difference and *r* is an irrational ratio. In Ref. [16], this phenomenon is called irrational phase synchronization (IPS). The numerical discovery of IPS represents a significant extension to the current knowledge of phase synchronization, as it is common sense that for coupled periodic oscillators, the resonance tongues that correspond to the irrational frequency ratios are of zero measure. The question that naturally arises, therefore, is the following: can IPS be observed in a realistic system?

Motivated by the numerical discovery of IPS in Ref. [16], in the present work, we study IPS through experiments. Specifically, by coupling two nonidentical periodic metronomes, we demonstrate that IPS is, indeed, observable in realistic systems. Moreover, through numerical simulations of the corresponding model, it is found that in the IPS state, the system dynamics are weakly chaotic, i.e., the largest Lyapunov exponent is positive.

## Materials and Experimental Results

**Fig 1:**
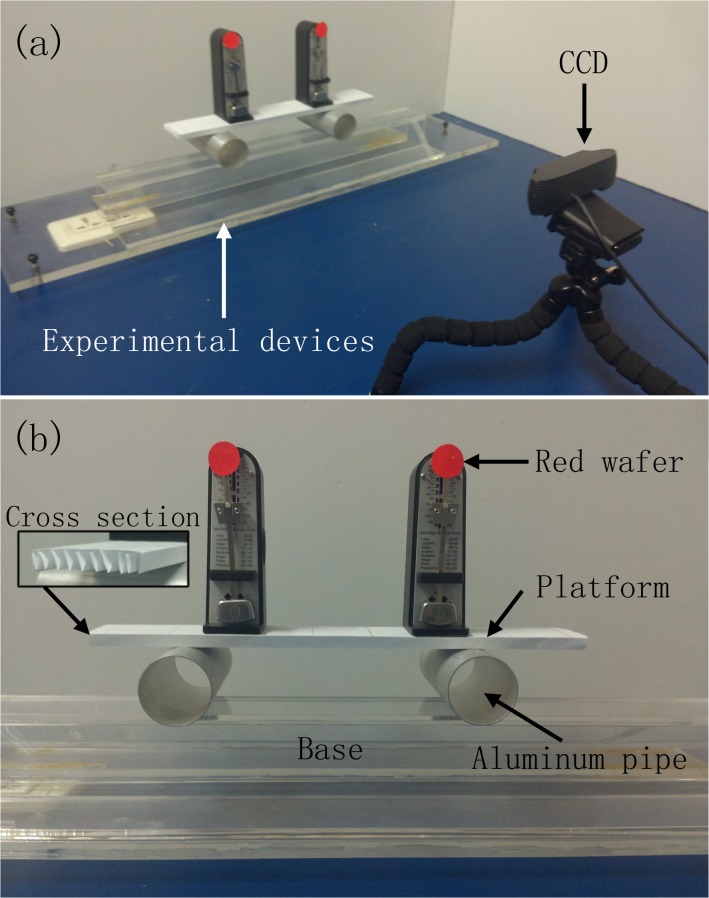
The experimental setup. (a) The experimental equipment and the CCD acquisition system. (b) The details of the experimental equipment.

The experiment setup employed in our studies is depicted in Fig. 1. Two metronomes are placed on a platform supported by two aluminum pipes. The specific metronome used is the Taktell Piccolino (Series 890) manufactured by Wittner GmbH & Co. KG, Germany, which has a mass of 94 g and whose energy is supplied by a manual winding spring. By adjusting the position of the bob on the pendulum, the frequency of the metronome can be changed continuously from 40 (largo) to 208 (prestissimo) beats per minute (BPM). The platform underneath the metronomes is formed of two sheets of A4 paper folded in a zigzag pattern. The mass of the platform is approximately 4.366 g. Two identical aluminum pipes with inner and external diameters of 39 mm and 41 mm, respectively, are placed in parallel below the platform. Because the motion of the pendulum bob is perpendicular to the pipe axes, a bidirectional coupling between the metronomes is established through the platform. Because the platform is light and the diameter of the pipes is large, the coupling between the metronomes is relatively strong. Underneath the pipes is placed a horizontal support that serves as the base of the experiment.

The motions of the metronomes are monitored by a charge-coupled device (CCD) acquisition system, and the recorded data are analyzed using computer software [17,18]. To facilitate the recording process, we affix a red wafer to the top of the pendulum on each metronome. This slightly increases the mass of the metronome (by approximately 0.01 g) and, consequently, slightly decreases the frequency of the metronome. The metronome device, the acquisition system, and the data analysis software have been developed and improved through a series of studies and have proven to be stable and reliable in various experiments [17,18,19]. The metronome device was initially designed by Zou et al. in Ref. [19] and was later improved on by several other researchers [20,21]. In Ref. [20], it is shown that by varying the coupling strength and the frequency mismatch, the metronomes can achieve phase or envelope synchronization. In Ref. [21], it is further demonstrated that by varying the system parameters, the basin of the phase synchronization can be significantly modified.

**Fig 2:**
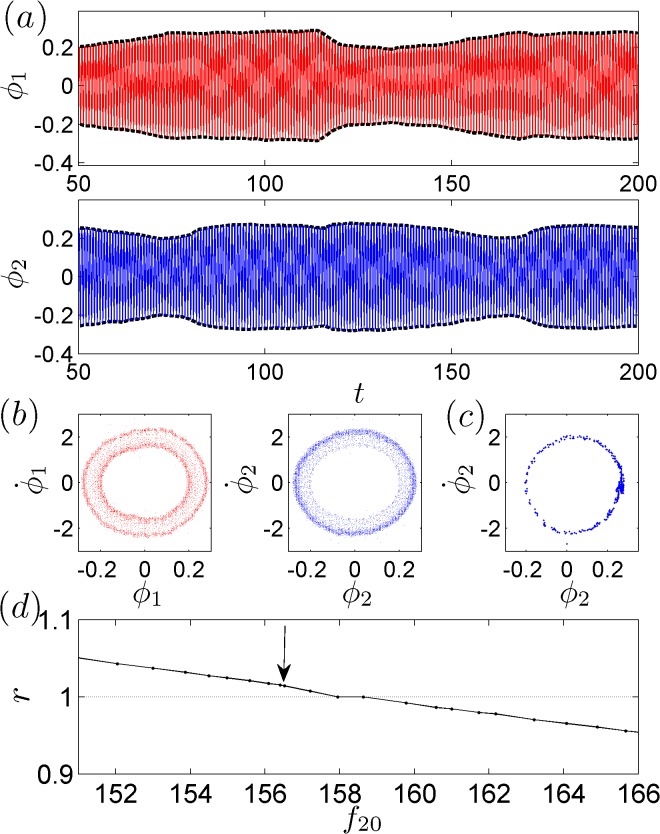
The experimental results. For *f*_10_ = 158.47 BPM and *f*_20_ = 156.52 BPM, we present (a) the time evolutions of *ϕ*_1,2_, where envelopes of *ϕ*_1,2_ exhibit anti-phase synchronization; (b) the phase trajectory in the $\left(\phi ,\dot{\phi }\right)$ space; and (c) the Poincaré maps. (d) The variation of the phase-locking ratio, *r*, as a function of the natural frequency of the second metronome, *f*_20_. The arrow denotes the point at which *r* = *π* / 3.1.

In our experiments, we fix the frequency of the first metronome to 160 BPM while varying the frequency of the second metronome gradually from 152 to 168 BPM. Note that the attachment of the wafer shifts the natural frequency of the first metronome to *f*_10_ = 158.47 BPM. With the natural frequency of the second metronome chosen to be *f*_20_ = 156.52 BPM, we plot in Fig. 2A the time evolution of the two phases, *ϕ*_1_(*t*) and *ϕ*_2_(*t*). The envelopes of *ϕ*_1_ and *ϕ*_2_ exhibit the phenomenon of anti-phase synchronization [20]. To provide further details on the dynamics of the phases, in Fig. 2B, we present the system trajectory in the $\left(\phi_{1,2},{\dot{\phi }}_{1,2}\right)$ spaces. It is observed that the trajectory is irregular in both spaces. Using the Poincaré surface-of-section method (i.e., by recording *ϕ*_2_ at the moments when ${\dot{\phi }}_{1}=0$ and ${\ddot{\phi }}_{1}<0$), we replot in Fig. 2C the discrete evolution of the system trajectory in the $\left(\phi_{2},{\dot{\phi }}_{2}\right)$ space, which reveals that the system dynamics are chaotic. A careful evaluation of the time evolution of *ϕ*_1,2_ also indicates that |Δ_*r*_ *ϕ − c*| < *π*, with *r* = *π* / 3.1 (the details of how to determine this irrational ratio are presented in the next section). That is, the two metronomes achieve the IPS state. To validate the experimental findings, we gradually vary the natural frequency of the second metronome from 152 to 168 BPM and investigate the synchronization behaviors of the two phases. The experimental results are presented in Fig. 2D. It is observed that as *f*_20_ increases, the phase-locking ratio, *r*, varies continuously. In particular, near the testing frequency *f*_20_ = 156.52, *r* continuously decreases as *f*_20_ increases. According to Ref. [16], given that *r* is continuously varying over some interval of the parameter space, IPS can always be achieved through careful tuning of the parameter within this interval.

## Methods and Numerical Results

**Fig 3:**
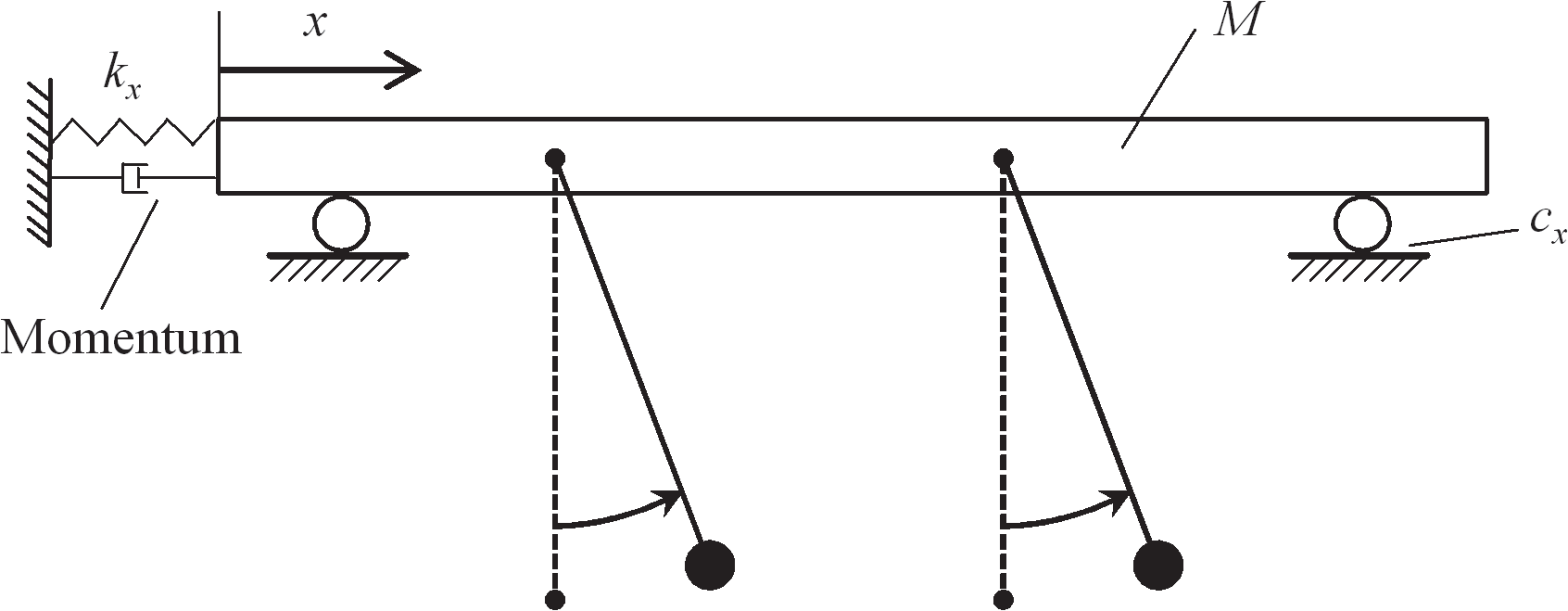
A schematic illustration of the simplified model.

To confirm the experimental findings, we next study IPS using numerical simulations based on a simplified model. By treating the metronomes as self-sustained periodic oscillators and regarding the platform as being driven by a liner spring, the experimental setup depicted in Fig. 1 can be simplified to the model schematically illustrated in Fig. 3 [22,23]. Based on this simplified model, the phase dynamics of the metronomes, *ϕ*_1,2_(*t*), and the displacement of the platform, *x*(*t*), can be calculated using the following Euler-Lagrange equations (please refer to Ref. [18] for further details regarding how to derive the equations from the simplified model):

$$m_{i}l_{i}^{2}{\ddot{\phi }}_{i}+m_{i}\ddot{x}l_{i}\;\text{cos}\phi_{i}+c_{\phi_{i}}{\dot{\phi }}_{i}+m_{i}gl_{i}\;\text{sin}\phi_{i}=M_{D_{i}},i=1,2,\ldots ,N$$ (1)

$$\left(M+\sum_{i=1}^{N}m_{i}\right)\ddot{x}+c_{x}\dot{x}+k_{x}x+\sum_{i=1}^{N}\left(m_{i}l_{i}{\ddot{\phi }}_{i}\;\text{cos}\phi_{i}-m_{i}l_{i}{\dot{\phi }}_{i}^{2}\;\text{sin}\phi_{i}\right)=0$$ (2)

Where *i* = 1,2 is the metronome index. In Eq. (1) (the equation for the phase dynamics), *m*_*i*_ is the mass of the pendulum bob, *l*_*i*_ is the pendulum length, *c_ϕ_i__* is the damping coefficient, and *g* ≈ 9.8 is the gravitational acceleration. *M_D_i__* is the external energy supplied by the metronome escapement, which is used to compensate for the energy dissipated by friction. The external energy has the form

$$M_{D_{i}}=\{\begin{array}{ll} 0.075m_{i}, & 0<\phi_{i}<\theta \;\text{and}\;{\dot{\phi }}_{i}>0,\\ -0.075m_{i}, & -\theta <\phi_{i}<0\;\text{and}\;{\dot{\phi }}_{i}<0,\\ 0, & \text{otherwise}. \end{array}$$ (3)

where *θ* is a small angle (*π* / 36 rad) below which the escapement is activated. In Eq. (2) (the equation for the platform displacement), *M* is the total mass of the two metronomes and the paper platform, *c*_*x*_ is the friction of the pipes, and *k*_*x*_ is the linear damping of the beam.

For consistency with the experimental setup, we set the (dimensionless) parameters in the modeling equations as follows: *m*_1_ = *m*_2_ = *m* = 1, *c*_*ϕ*_ = 0.01, *M* = 30, *θ* = *π* / 36, *c*_*x*_ = 1.5, and *k*_*x*_ = 1. By setting *l*_1_ = 0.014248, we specify *f*_10_ = 160 BPM for the first metronome. The natural frequency of the second metronome can be modified continuously by adjusting *l*_2_, following the relation *l*_2_ ≈ *g*(60/*πf*_20_)^2^. For the simulations, we set the initial conditions to ${\dot{\phi }}_{1,2}(t=0)=0$ and $x(t=0)=\dot{x}(t=0)=0$ and choose *ϕ*_1,2_(*t* = 0) randomly within the range (−*π* / 2,*π* / 2). Eqs. (1) and (2) are integrated using the 4th-order Runge-Kutta method with a time step of Δ*t* = 0.0001. In our simulations, the working frequencies, *f*_1,2_, of the metronomes are obtained by averaging over a period of *t* = 1 × 10^4^. (Please note the difference between the working frequencies, *f*_1,2_, and the natural frequencies, *f*_10,20_, of the metronomes. The former characterizes the coupled metronomes, whereas the latter describes the isolated metronomes.) The first question we are interested in is the following: can the working frequencies of the metronomes become locked into an irrational ratio, i.e., *r*′ = *f*_1_ / *f*_2_, where *r*′ is an irrational number? (The locking of the working frequencies into an irrational ratio is a necessary condition for the observation of IPS.)

**Fig 4:**
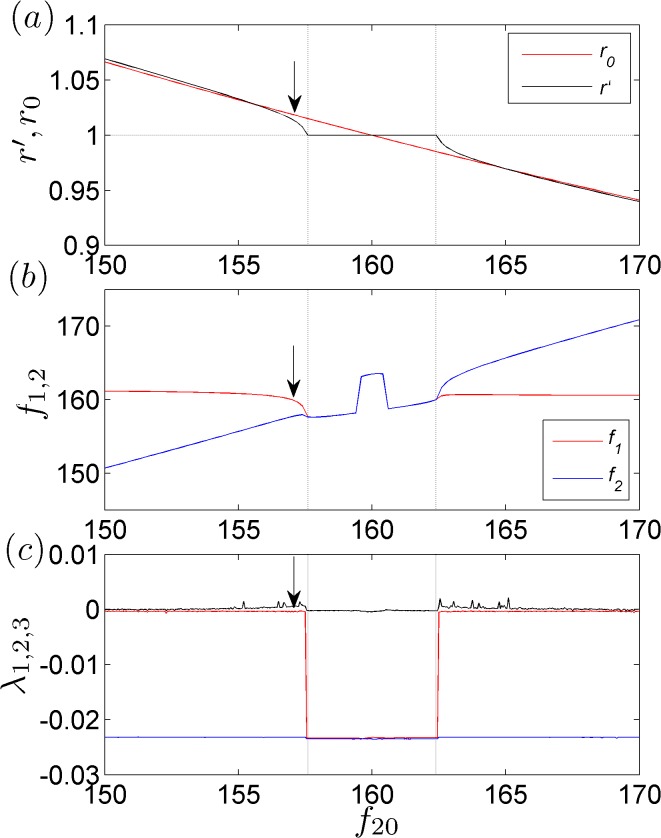
The numerical results. (a) The variation in the ratio of the working frequencies, *r*′ = *f*_1_ / *f*_2_, as a function of *f*_20_ for *f*_10_ = 160 BPM. As a reference, the ratio of the natural frequencies, *r*_0_ = *f*_10_ / *f*_20_, is also plotted. (b) The variations in the working frequencies, *f*_1,2_, as functions of *f*_20_. Resonance-like behavior is observed in the region *f*_20_ ∊ [160 − 2.4,160 + 2.4]. (c) The variations in the Lyapunov exponents, *λ*_1,2,3_, as functions of *f*_20_. At *f*_20_ = 157.08915, the working frequencies are locked into the ratio *r*′ = *π* / 3.1, and the largest Lyapunov exponent is *λ*_1_ ≈ 0.001.

To construct a global picture of the locking of the working frequencies, we first consider the variation of *r*′ as a function of *f*_20_. The results are presented in Fig. 4A. It is observed that with the exception of the interval [*f*_10_ − 2.4,*f*_10_ + 2.4], where *r*′ = *f*_1_ / *f*_2_ = 1, the value of *r*′ is continuously decreasing as the increase of *f*_20_. This behavior of *r*′ is consistent with the experimental results presented in Fig. 2D. To acquire further details on the locking of the two working frequencies, we then investigate the variations of *f*_1,2_ as functions of *f*_20_. The results are plotted in Fig. 4B. It is observed that as *f*_20_ increases, both *f*_1_ and *f*_2_ vary continuously. This finding confirms the continuous variation in *r*′ observed in Fig. 3A. Interestingly, in Fig. 4B, it is also observed that within the interval [*f*_10_ − 2.4,*f*_10_ + 2.4], the locked frequencies (with the locking ratio *r* = 1) are largely divergent from the natural frequency of the first metronome, *f*_10_. Specifically, *f*_1,2_ > *f*_10,20_ holds in the interval [*f*_10_ − 0.4,*f*_10_ + 0.4], and *f*_1,2_ < *f*_10,20_ holds in the intervals [*f*_10_ − 2.4,*f*_10_ − 0.4] and [*f*_10_ + 0.4,*f*_10_ + 2.4]. This frequency fluctuation behavior may be induced by the resonance of the metronome oscillation with the platform motion, and this possibility will be investigated in our future studies. To exclude the possibility of quasiperiodic motion, we also calculate the variations of the Lyapunov exponents, *λ*_1,2,3_, as functions of *f*_20_, based on the algorithm introduced in Refs. [24,25]. The results are plotted in Fig. 4C. It is observed that the largest Lyapunov exponent, *λ*_1_, is equal to 0 within the region [*f*_10_ − 2.4,*f*_10_ + 2.4] and is positive in the other regions. Fig. 4 thus implies that in the regions *f*_20_ < *f*_10_ − 2.4 and *f*_20_ > *f*_10_ + 2.4, the working frequencies of the metronomes can become locked into irrational ratios and the system dynamics are chaotic.

**Fig 5:**
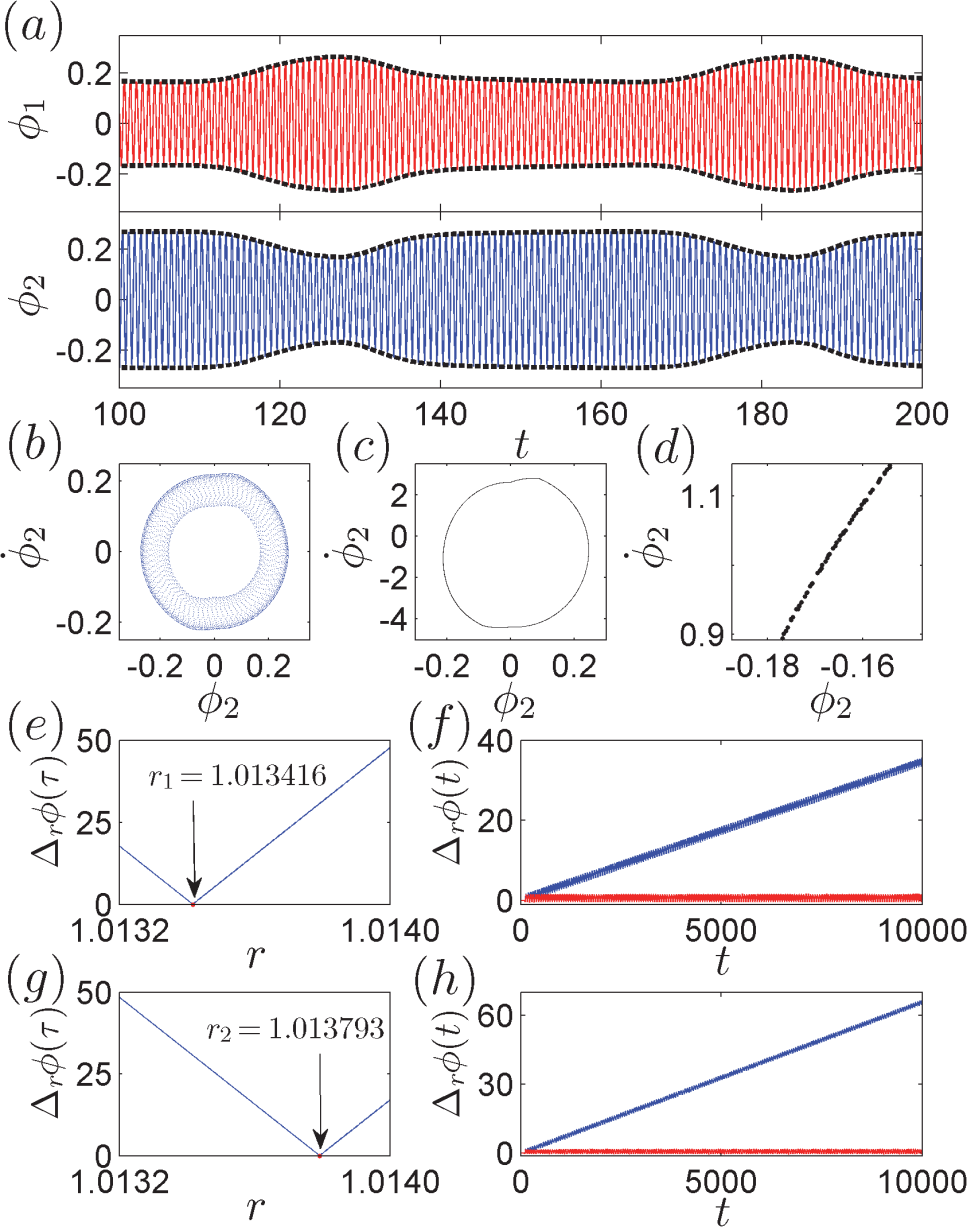
Examples of IPS observed in numerical simulations. For *f*_10_ = 160 BPM and *f*_20_ = 157.08915, we present (a) the time evolution of the phases, *ϕ*_1,2_, where anti-phase synchronization is observed in the phase envelopes; (b) the phase trajectory in the $\left(\phi_{2},{\dot{\phi }}_{2}\right)$ space; (c) the Poincaré maps; and (d) a magnified view of the Poincaré maps. (e) The variation of the phase difference Δ_*r*_ *ϕ* at *t* = 1 × 10^4^ as a function of the phase ratio *r*. Δ_*r*_ *ϕ* reaches a minimum as r approaches *r*_1_ = *π* / 3.1. (f) The time evolution of Δ_*r*_ *ϕ* for *r* = *r*_1_ (red) and *r* = *r*_1_ − 4.17 × 10^−4^ (blue). (g) The variation of the phase difference Δ_*r*_ *ϕ* at *t* = 1 × 10^4^ as a function of *r* near $r_{2}=\sqrt{37}/6$. (f) The time evolution of Δ_*r*_ *ϕ* for *r* = *r*_2_ (red) and *r* = *r*_1_ − 7.93 × 10^−4^ (blue).

Setting *f*_20_ = 157.08915, we now investigate the evolutional dynamics of the phases in detail. From Fig. 4A and B, we know that in this case, the phases will be locked into the ratio *r*_*c*_ ≈ *π* / 3.1, and from Fig. 4, we also know that the system dynamics are chaotic, with *λ* ≈ 0.001. In Fig. 5A, we plot the time evolution of the two phases *ϕ*_1,2_. Consistent with the experimental results [Fig. 2A], the envelopes of *ϕ*_1_ and *ϕ*_2_ exhibit the phenomenon of anti-phase synchronization. Applying the same method used in the experimental analysis, in Fig. 5B, 5C and 5D, we present the phase trajectory in the $\left(\phi_{2},{\dot{\phi }}_{2}\right)$ space, the Poincaré maps and a magnified view of the latter. Consistent with the observation of the positive Lyapunov exponent, the phases exhibit chaotic motion [26].

Can the phases also become locked into an irrational ratio? Because of the chaotic nature of the system dynamics, the locking of the working frequencies does not guarantee the same locking of the phases. Furthermore, because of the limited computational precision, it is difficult to judge whether the phases could become locked exactly into a truly irrational ratio or merely into a decimal ratio with a finite number of digits. To address this question, an effective approach is to investigate the variation of the phase difference Δ_*r*_ *ϕ* (t) = |*ϕ*_*1*_ *− rϕ*_*2*_| as a function of *r* in the neighboring region of the irrational ratio of interest. If the phase difference Δ_*r*_ *ϕ* gradually approaches 0 as *r* approaches the irrational ratio *r*_1_, then one can conclude that at *r*_1_, the two metronomes achieve the state of IPS [16]. Using this method, we plot in Fig. 5E the variation in Δ_*r*_ *ϕ* (obtained at the instant *t* = 1×10^4^ of the system evolution) as a function of *r* in the range *r* ∊ [1.1032,1.0140]. It is clearly observed that Δ_*r*_ *ϕ* approaches 0 as *r* approaches *r* = *π* / 3.1 ≈ 1.013417. As depicted in Fig. 5F, the value of Δ_*r*_ *ϕ* remains equal to 0 for *r* = *r*_1_ but is monotonically increasing when *r* = *r*_1_ − 4.17×10^−4^.

Fig. 5G and H illustrate another example of IPS. In Fig. 5G, we change the natural frequency of the second metronome to *f*_20_ = 157.06244 and plot the variation of the phase difference Δ_*r*_ *ϕ* as a function of *r* within the same range used in Fig. 5E. As *r* approaches $r_{2}=\sqrt{37}/6\approx 1.013793$, Δ_*r*_ *ϕ* monotonically decreases to 0. Again, in Fig. 5H, it is observed that Δ_*r*_ *ϕ* remains equal to 0 when *r* = *r*_2_ and monotonically increases for *r* = *r*_2_ − 7.93×10^−4^. By calculating the largest Lyapunov exponent, it is shown that the system is chaotic for *f*_20_ = 157.06244, as *λ*_1_ > 0. We thus conclude from Fig. 5E-5H that IPS can indeed be achieved between the coupled metronomes, given that the natural frequencies of the metronomes are properly chosen, e.g., (*f*_10_,*f*_20_) = (160,157.08915) (*r* = *π* / 3.1) or (*f*_10_,*f*_20_) = (160,157.06244) $\left(r=\sqrt{37}/6\right)$.

## Discussion

A few remarks regarding the present work are in order. First, although it is impossible for the phases of two coupled oscillators to be locked exactly into an irrational ratio in practice, our studies show that the impossibility of this phenomenon is attributable only to limited experimental and numerical precision. In other words, through improvements to the experimental and numerical precision, IPS can be gradually approached in realistic systems. This fact can be partially understood from the results presented in Fig. 5E and 5F, where the phase difference Δ_*r*_ *ϕ* gradually decreases to 0 as *r* approaches *r*_*c*_. Second, we would like to note that in the present work, although the IPS state is chaotic, the motion of the isolated metronomes is, in fact, periodic. The underlying mechanism of these chaotic dynamics is still unknown to us. Therefore, it will be important to confirm whether IPS can be experimentally realized in coupled chaotic oscillators. Third, in numerical studies, we have observed resonance-like behavior in the locked working frequencies, i.e., *r*′ = 1 near *f*_20_. The dynamical mechanism is also unknown to us. Finally, considering the fact that many realistic systems consist of many coupled oscillators, it will be intriguing to investigate whether IPS can be observed in spatially extended systems, e.g., three oscillators coupled in a ring structure. We hope to address these questions in our future studies.

To summarize, we have experimentally studied IPS in coupled nonidentical metronomes. We find that when the natural frequencies of the metronomes are properly chosen, the phases of the two metronomes can become locked into irrational ratios. The experimental findings are verified by numerical simulations based on a simplified model. Our studies suggest that IPS is not merely of theoretical interest but also of practical concern.
